# Binarization Mechanism Evaluation for Water Ingress Detectability in Honeycomb Sandwich Structure Using Lock-In Thermography

**DOI:** 10.3390/ma15062333

**Published:** 2022-03-21

**Authors:** Yoonjae Chung, Ranjit Shrestha, Seungju Lee, Wontae Kim

**Affiliations:** 1 Eco-Sustainable Energy Research Institute, Kongju National University, 1223-24 Cheonan-daero, Seobuk-gu, Cheonan-si 31080, Korea; dbswosla79@kongju.ac.kr; 2 Department of Mechanical Engineering, School of Engineering, Kathmandu University, Dhulikhel, Kathmandu 45200, Nepal; ranjit.shrestha@ku.edu.np; 3 Department of Future Convergence Engineering, Kongju National University, 1223-24 Cheonan-daero, Seobuk-gu, Cheonan-si 31080, Korea; cow123456798@smail.kongju.ac.kr; 4 Division of Mechanical & Automotive Engineering, Kongju National University, 1223-24 Cheonan-daero, Seobuk-gu, Cheonan-si 31080, Korea

**Keywords:** water ingress, non-destructive testing (NDT), lock-in thermography (LIT), composites, signal processing, image processing, Otsu algorithm, binarization, contrast to noise ratio (CNR)

## Abstract

The growing use of composite honeycomb structures in several industries including aircraft has demonstrated the need to develop effective and efficient non-destructive evaluation methods. In recent years, active thermography has attracted great interest as a reliable technology for non-destructive testing and evaluation of composite materials due to its advantages of non-contact, non-destructive, full-area coverage, high speed, qualitative, and quantitative testing. However, non-uniform heating, low spatial resolution, and ambient environmental noise make the detection and characterization of defects challenging. Therefore, in this study, lock-in thermography (LIT) was used to detect water ingress into an aircraft composite honeycomb sandwich structure, and the phase signals were binarized through the Otsu algorithm. A square composite honeycomb with dimensions of 210 mm × 210 mm along with 16 different defective areas of various sizes in groups filled with water by 25%, 50%, 75%, and 100% of the cell volume was considered. The sample was excited at multiple modulation frequencies (i.e., 1 Hz to 0.01 Hz). The results were compared in terms of phase contrast and CNR according to the modulation frequency. In addition, the detectability was analyzed by comparing the number of pixels of water ingress in the binarized image and the theoretical calculation.

## 1. Introduction

A carbon fiber reinforced plastic (CFRP) laminate having a strong anisotropy consists of a sandwich structure. The core of this structure is sandwiched between the outer skins on both sides. It offers excellent strength to weight ratios and is mainly used in the aviation industry, specifically in aircraft flooring, doors, wing flaps and rudders; automobiles; and building panels [1]. It is used as a honeycomb sandwich panel in which a CFRP skin is bonded to a honeycomb core [2,3,4].

Defects in the sandwich structure occur due to various causes. Aircraft are often damaged after the commencement of operations [5]. Honeycomb composites are hollow structural materials, making them sensitive to liquid intrusion. Water and hydraulic oil are common types of liquid intrusion that can be caused by poor sealing or skin damage [6].

Numerical analysis and non-destructive testing are applied as methods for preventing and maintaining critical structural defects. Many numerical methods have been proposed in previous studies to predict the nucleation and propagation of failure in honeycomb structures [7]. The purpose of these studies is to develop a phase field model that can predict both the defect propagation path and the mechanical response of fiber-reinforced composite laminates [8]. Alternatively, to detect the presence of water in the aircraft structure early before fatal damage due to this problem, there has been considerable interest in various non-destructive testing (NDT) methods in the field, and research has been conducted in this area [9]. Over the years, ultrasonic [10], radiological, and neutron radiography, helium mass spectrometer leak detection, and hot water leak testing techniques have proven to be effective in controlling and detecting the water ingress inside honeycomb structures [11]. However, the ultrasonic inspection takes a lot of inspection time due to the inspection for each point, and the productivity is very low. Additionally, radiation and neutron radiographic examination techniques are rarely used in the field due to complex problems such as size, double-sided installation, and strict radiation precautions. Hot water leak testing has the disadvantage of potentially allowing water to penetrate components, and helium mass spectrometry techniques tend to be expensive to process and significantly dependent on user experience. In this context, infrared thermal imaging (IRT) has very great advantages in that it is fast, non-contact, non-destructive, has a fast inspection speed, large inspection area, is safe, and is portable.

IRT collects and analyzes thermal information using an infrared camera, a non-contact thermal imaging device based on the fact that all objects with an absolute temperature of 0K emit infrared energy. The infrared energy detected by the infrared camera is converted and can be processed to create a thermal image. IRT is generally divided into two approaches: passive thermography and active thermography [12]. LIT, also known as modulated thermal image, is an advanced NDT technology in which an object being inspected is heated with modulated thermal energy, causing the surface temperature to change periodically [13,14]. Basic concept and theory for the LIT have been frequently mentioned through previous literature [15,16,17,18]. The application of IRT NDT technology in the field of aircraft was first proposed in the 1960s [19]. In the 1990s, pulsed thermography was applied to detect corrosion and detachment of B737 testbed aircraft [20,21]. Since then, various IRT methods and data processing algorithms have been developed and applied to the detection of water ingress on honeycomb sandwich composites. Previous studies have been conducted to quantify the water ingress and defects such as delamination, impact damage, and fatigue failure on sandwich composites and structures [22,23,24]. In addition, it has been reported that PT and LIT were compared and that the phase angle of LIT produced a higher signal-to-noise ratio than PT [25]. PT is more susceptible to non-uniform heating, surface emissivity changes, surface morphology, and noise than LIT. The eddy current thermography has been introduced as another IRT technique to detect and characterize defects in honeycomb sandwich panels [26]. Furthermore, vibration and IRT combined neural networks have also proposed a technique for identifying the type, location, and extent of damage on sandwich composites [27]. Recently, the LIT algorithm was applied to test the water ingress of honeycomb sandwich panels, and SNR improvement studies were conducted [14].

This study presents an experimental study of the process of detecting water ingress in a typical composite honeycomb sandwich panel using LIT. The main purpose of this study was to perform a quantitative evaluation of LIT in reference samples under different conditions with various sizes and water ingress rates. Typically, a four-point algorithm is used to process LIT experimental data. The second purpose is to process LIT experimental data using binarization and compare its performance with an Otsu algorithm in terms of water ingress content. This study also explored the influence of water ingress content as well as the modulation frequency obtained with the method above-mentioned. Finally, the relationship between phase contrast and contrast to noise ratio (CNR) and its effect on the binarized image is presented. The remainder of the paper is composed as follows. In Section 2, the basic concept of LIT is provided. Subsequently, the theory of the four-point method and Otsu algorithm is provided in Section 3. In Section 4, the details of the test sample and experimental process are described. Section 5 presents the experimental results, and the conclusions of this study are indicated in Section 6.

## 2. Lock-In Thermography (LIT)

LIT is typically applied to amplitude and phase measurements to evaluate underlying defects. The phase and amplitude information calculated by the processing of the thermal images recorded for each pixel are stored in the form of a 2D matrix, which is then converted to images known as phase and amplitude images. The amplitude image shows the total temperature rise of the system during the power cycle and the phase image provides the power to the device and the time delay between subsequent heating of the surfaces. LIT is a technique to obtain the amplitude and phase images for quantitatively analyzing the size and depth of defects.

The periodical heat transfer through the semi-infinite (planar) body resulting in the thermal wave can be expressed by Equation (1) [28,29].

(1) $$T_{z,t}=A\left(z\right)\cos\left[\omega t-\emptyset \left(z\right)\right]=T_{0}e^{-\frac{z}{\mu }}\cos\;\left[\omega t-\frac{2\pi z}{\lambda }\right]$$

where A(z) resembles the thermal amplitude; ω=2πf [rad/s] is expressed as an angular frequency; t is the time; ∅(z) is the phase shift; T_0_ [°C] is an initial change in temperature due to a heat source; z [mm] is the defect depth; λ=2πμ [m] is expressed as the thermal wavelength; and μ [m] is the thermal diffusion length and can be calculated from the thermal diffusivity and the modulation frequency using Equation (2) [28,30].

(2) $$\mu =\sqrt{\frac{2\alpha }{\omega }}=\sqrt{\frac{\alpha }{\pi f}}$$

where f [Hz] is the modulation frequency.

Equation (2) revealed that the thermal diffusion length depends on the modulation frequency and thermal diffusivity (i.e., thermophysical properties: thermal conductivity, density, and specific heat capacity) of the material.

Considering Equation (1), the phase shift ∅(z) can be defined by Equation (3), which indicates that the phase is associated with defect depth (z) without the influence of other parameters [15].

(3) $$\emptyset =\frac{z}{\mu }$$

In contrast, amplitude A(z) can be defined by Equation (4), which indicated that amplitude is associated with the initial temperature $T_{0}$ and moreover to some other parameters [28].

(4) $$A\left(z\right)=T_{0}e^{-\frac{z}{\mu }}$$

## 3. Image Processing

### 3.1. Phase and Amplitude Computation

For the experimental LIT data, the four-point method and the Fourier transform computes the amplitude and phase angle data from the recorded temperature–time history of each pixel, then stored in the form of 2D matrices and converted to amplitude and phase images. Amplitude and phase images are related to the thermal diffusivity and the propagation time, respectively.

### 3.2. Four-Point Method

**Figure 1:**
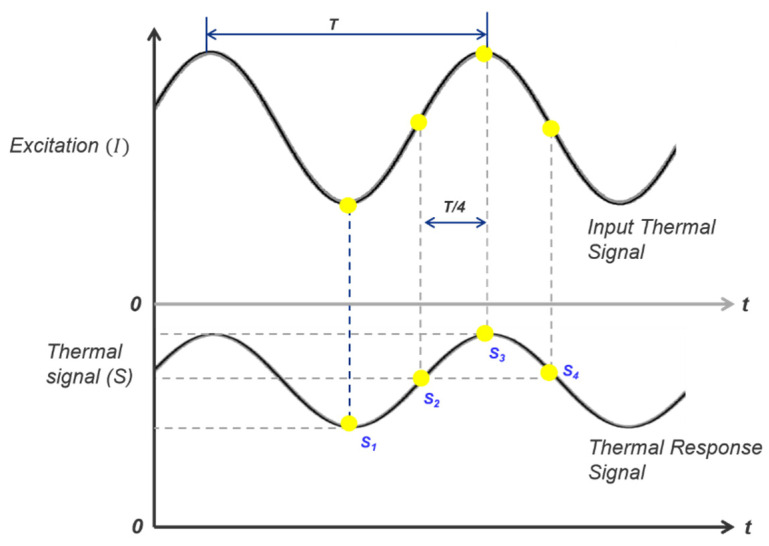
Principle of the four-point method in lock-in thermography.

If S_1_, S_2_, S_3_, and S_4_ are four equidistant thermal images, as shown in Figure 1, in a constant period, then the phase (∅) and amplitude (A) images can be expressed by Equations (5) and (6), respectively [31,32].

(5) $$\emptyset =\tan^{-1}\left(\frac{S_{1}-S_{3}}{S_{2}-S_{4}}\right)$$

(6) $$A=\sqrt{{\left(S_{1}-S_{3}\right)}^{2}+{\left(S_{2}-S_{4}\right)}^{2}}$$

The four-point method is fast and valid for sinusoidal stimulation, but is affected by noise. The increased number of cycles results in the denoising of the signal.

### 3.3. Binarization Using the Otsu Algorithm

There are many techniques to analyze thermal images, but the simplest and easiest method among them is to use a threshold to binarize the image. Through binarization, automated detection can be characterized for various defects. This is used in the image pre-processing stage to separate the background from the image opening to only extract pixels with data (brightness, etc.) above a certain level, or to simplify the entire image information [33].

In the Otsu algorithm, the threshold is calculated based on the gray scale. An image is divided into two using an image histogram, and an optimal threshold value can be estimated. In the binary image, [0, k] is classified as ‘class 1’, and [k, 1] is classified as ‘class 2’ based on the threshold value k. Through this process, binary images can be acquired based on a threshold value. In general, converting a 2D thermal image to a binary image can characterize the characteristics of the defects [34,35].

In order to classify into two binary images, it is necessary to calculate an optimal threshold value. If it is an M × N image with L intensity levels such as 0, 1, 2, …, L − 1, pixels with intensity values within [0, k] are classified as class 1, and intensity values within [k + 1, L + 1] are classified as class 2. The probability that a pixel is classified into class 1 or 2 is defined by Equations (7) and (8) [36]:

(7) $$P_{1}\left(k\right)=\sum_{i=0}^{k}P_{i}$$

(8) $$P_{2}\left(k\right)=1-P_{1}\left(k\right)$$

The average intensity values of pixels classified into classes 1 and 2 are defined as Equations (9) and (10).

(9) $$m_{1}\left(k\right)=\frac{1}{P_{1}\left(k\right)}\sum_{i=0}^{k}{\mathrm{iP}}_{i}$$

(10) $$m_{2}\left(k\right)=\frac{1}{P_{2}\left(k\right)}\sum_{i=k+1}^{L-1}{\mathrm{iP}}_{i}$$

There are mean intensity values up to the k level, which for all images is

(11) $$m_{G}=P_{1}m_{1}+P_{2}m_{2}$$

In order to calculate the optimal threshold value, the Otsu algorithm should allow the concept of between-class variance. The equation of between-class variance is defined as Equation (12).

(12) $$\sigma_{b}^{2}=\frac{{\left(m_{G}P_{1}-m\right)}^{2}}{P_{1}\left(1-P_{1}\right)}$$

Calculating the optimal k value is a simple principle, although the optimal k value can be calculated only by substituting all k values in the intensity range [0, L − 1].

## 4. Methods and Materials

### 4.1. Test Sample

**Figure 2:**
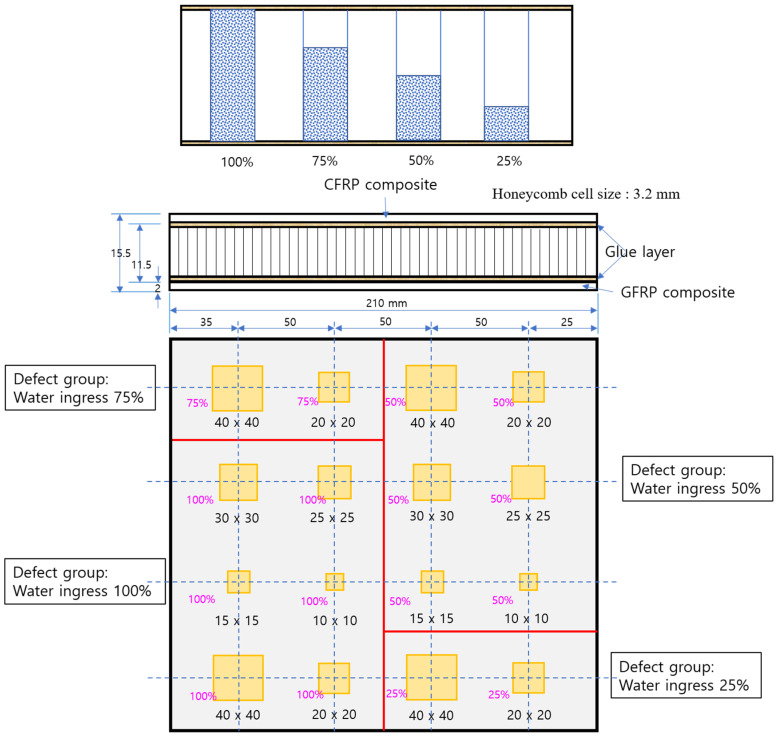
Schematic of the honeycomb-cored sandwich panel considering various water ingress conditions.

**Figure 3:**
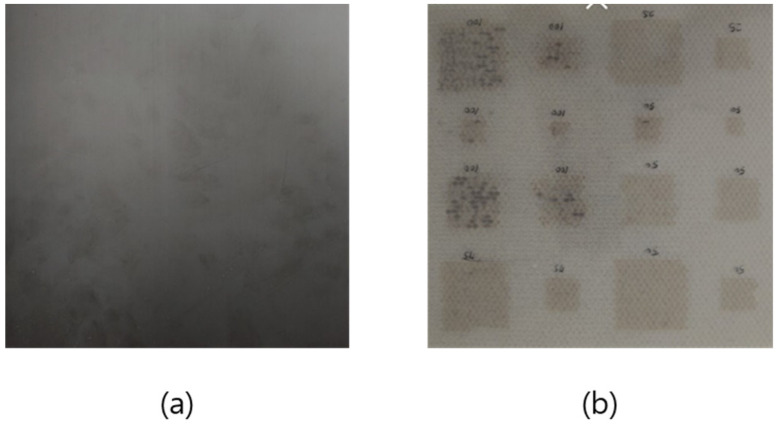
Aramid honeycomb-cored sandwich panel as a test sample. (**a**) The front face skin with CFRP and (**b**) the rear face skin with GFRP.

The study was conducted by applying a honeycomb sandwich sample fabricated by the EZ composite in Korea. The sample was applied with an aramid honeycomb core sandwich panel with CFRP on the front and glass fiber reinforced plastic (GFRP) composite face skin on the back. A heat-resistant phenolic resin was coated on the surface of the specimen to improve the strength and thermal properties. The CFRP skin on the front side of the sample was made of SKYFLEX WSN3KY and Mitsubishi Rayon TR-30, and the GFRP skin on the back side of the sample was made of GEP7628 and E-glass. Figure 2 and Figure 3 show the schematic diagrams and geometrical information of the honeycomb sandwich samples considering various water ingress conditions. The sample is a square plate with a size of 210 mm × 210 mm. In the sample, 16 types of water ingress under various conditions from 10 mm × 10 mm to 40 mm× 40 mm were considered. In addition, the water ingress rate was classified into four groups (filled with water by 25%, 50%, 75%, and 100% of the cell volume.)

### 4.2. Experimentation

**Figure 4:**
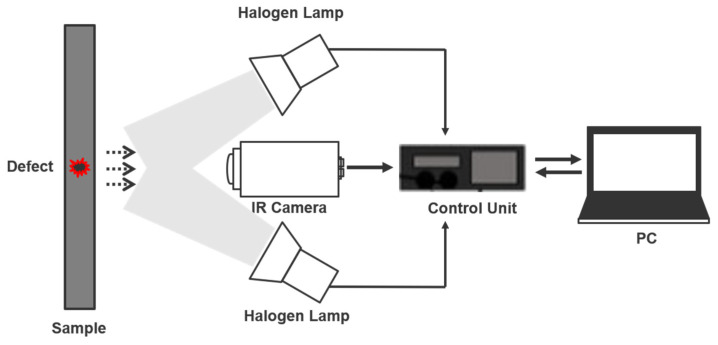
Experimental configuration of a lock-in thermography inspection system.

The experiment was conducted with lock-in thermography, which has light as a heat source from the outside. The surface of the sample was heated with a halogen lamp with a sinusoidal heat source. Thermal response from the surface due to the possession of a heat source was investigated with an infrared camera and temperature data were recorded. The heat source was controlled by power amplifiers and function generators and two lamps of 1 kW halogen were applied as external heat sources. The applied frequency was set by decreasing the frequency from 1 Hz to 0.01 Hz. The function generator (Agilent 33210A, Agilent Technologies, Santa Clara, CA, USA) was used to produce sinusoidal waves, and the infrared camera used to detect radiation from the surface of the specimen was applied with the FLIR SC645 model (640 × 480 pixels, 7.5 to 13 μm, Teledyne FLIR, Wilsonville, OR, USA). The sample can be placed on the infrared camera screen using a lens of 41.3 mm. Infrared images and temperature data were obtained using FLIR R&D commercial software. The frame of the camera was set at 50 frames per second for each frequency. The acquired data were processed through MATLAB 2021b software coding. A schematic of the general LIT test configuration is depicted in Figure 4.

## 5. Results and Discussions

### 5.1. Water Ingress Detectability in Phase Image

**Figure 5:**
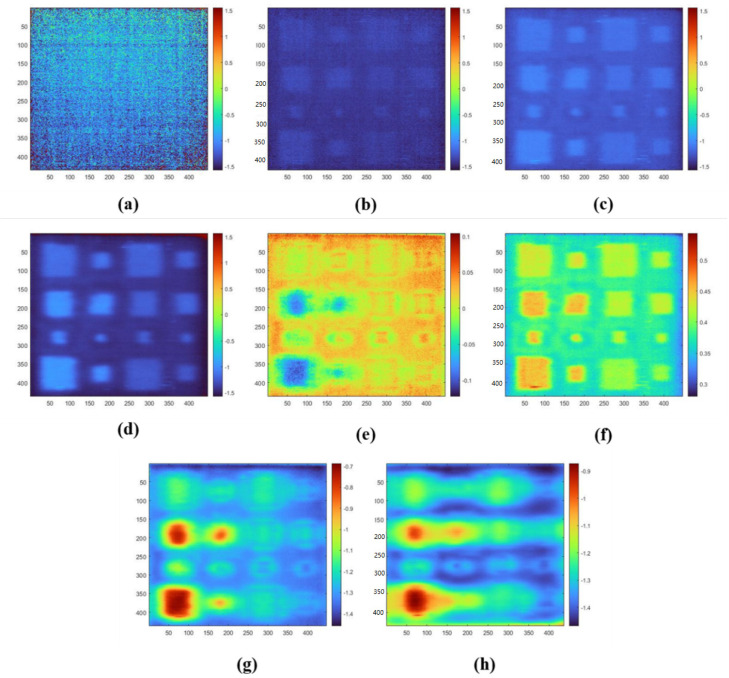
Phase images by the four-point method: (**a**) 1 Hz, (**b**) 0.5 Hz, (**c**) 0.2 Hz, (**d**) 0.1 Hz, (**e**) 0.07 Hz, (**f**) 0.05 Hz, (**g**) 0.03 Hz, (**h**) 0.01 Hz.

In this study, only the phase angle images obtained from the four-point method were considered in the analysis because phase angle images were relatively free from non-uniform heating, optical absorption, and emissivity variation when compared with the amplitude images. A series of thermal imaging experiments were conducted while reducing the modulation frequency from 1 Hz to 0.01 Hz. In the experiment, a total of two cycles of modulation frequency was applied, and all pixels of the thermal images were processed with phase through Equation (5). Figure 5 shows the resulting images of the phase images at 1 Hz, 0.5 Hz, 0.2 Hz, 0.1 Hz, 0.07 Hz, 0.05 Hz, 0.03 Hz, and 0.01 Hz.

**Figure 6:**
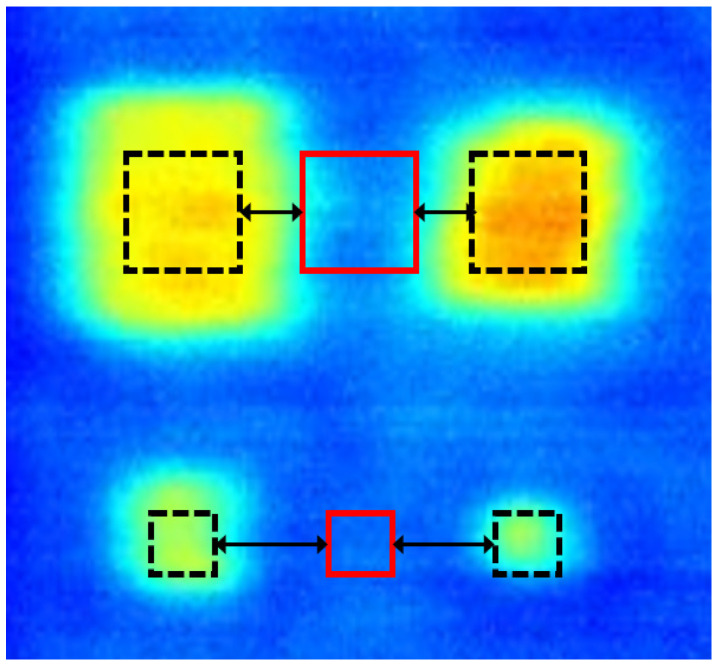
Representation of selected areas for contrast and CNR computation. The black dashed line enclosed area represents the water ingress areas and the red solid line enclosed area represents the sound areas.

This study shows that the possibility of detecting water ingress in the phase images depends on the applied modulation frequency. As shown in Figure 6, it can be seen that no water ingress was detected at the modulation frequency phase of 1 Hz. It can be seen that when the modulation frequency was reduced to 0.5 Hz, the contrast with the background was improved, and all water ingress began to be detected. In addition, as the modulation frequency was reduced to 0.03 Hz or less, the image resolution was lowered by moisture present therein, and the sharpness of defects deteriorated. This is due to strong external energy due to the different thermal conductivity between water and composites in the surface transient heat transfer mode.

### 5.2. Phase Contrast and CNR Trend

In LIT, it is important to analyze the effect of the modulation frequency of an external heating source. Therefore, in this study, the optimal frequency was determined through the phase contrast and noise contrast trends, and their relationship was analyzed. First, in order to determine the optimal modulation frequency, the contrast between the water ingress and the adjacent sound area was analyzed, and the contrast analysis is defined as Equation (13) [14].

(13) $$\Delta C=C_{d}-C_{s}$$

where $C_{d}$ is the phase angle of defective area (water ingress), and $C_{s}$ is the phase angle of the sound area.

D_1_, A_1_, A_3_, and D_3_ with fixed dimensions of 40 mm × 40 mm and water ingress rates of 100%, 75%, 50%, and 25%, respectively, were selected for analysis. As shown in Figure 6, for one, 20 × 20 pixels were applied for the water ingress and sound areas with dimensions of 20 mm × 20 mm or more, and 10 × 10 pixels were applied for the rest.

**Figure 7:**
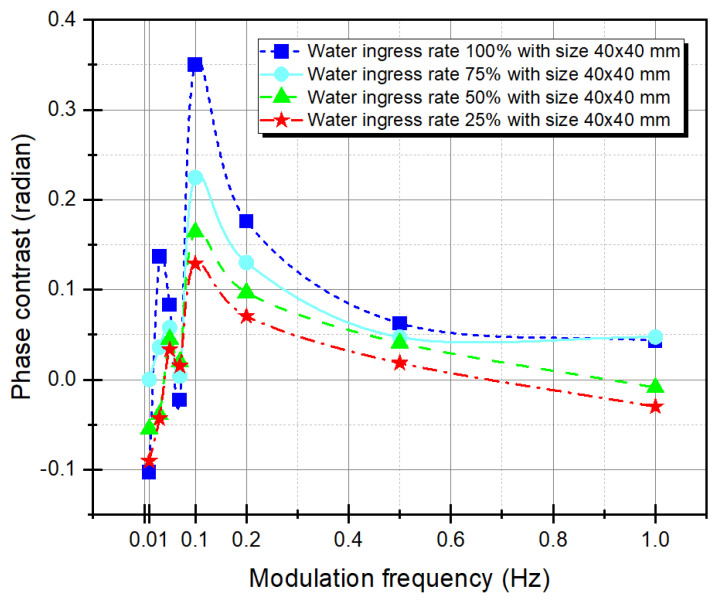
Phase contrast with the four-point method as a function of modulation frequency.

**Figure 8:**
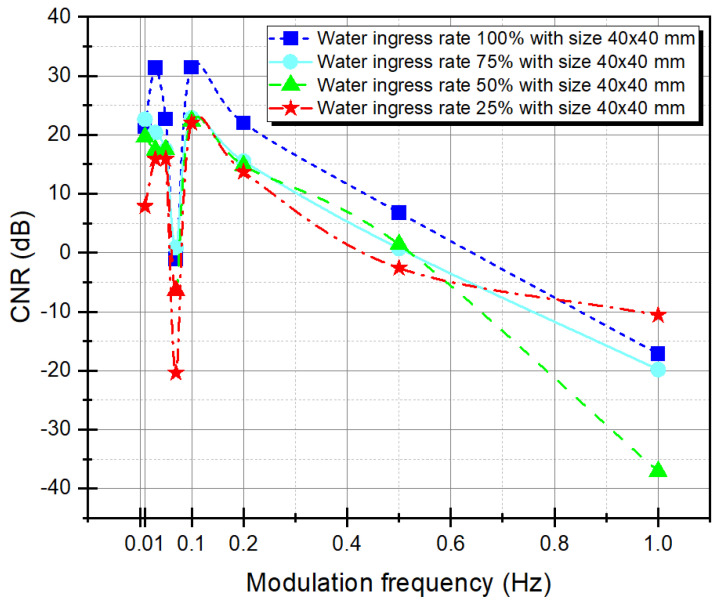
Phase-contrast with four-point method as a function of modulation frequency.

Figure 7 and Figure 8 show the phase contrast and CNR trends of D_1_, A_1_, A_3_, and D_3_, and Table 1 shows the total data of phase contrast and CNR. Figure 7 shows the phase contrast according to the modulation frequency. It can be seen that the phase contrast increases from 0.01 Hz to 0.1 Hz as the frequency increases, and then decreases again after 0.1 Hz. Therefore, phase detection can be estimated as the optimal frequency at 0.1 Hz. The maximum phase contrast of modulation frequency in 0.1 Hz, D_1_ was 0.3502 rad, A_1_ was 0.2247 rad, A_3_ was 0.1641 rad, and D_4_ was 0.1288 rad.

Second, to analyze the relationship between phase contrast and CNR, values according to the modulation frequencies were compared. CNR was the same as a signal to noise ratio (SNR) used in the electrical signal description. The CNR metric is used to differentiate the two regions of interest (ROI). CNR is the ratio of the size of the signal (defects) to the size of the noise (sound). Image processing is performed to increase the SNR, and the higher the CNR value, the better the detection ability of the fault. CNR is defined as Equation (14) [37,38].

(14) $$\mathrm{CNR}=20\log_{10}\left(\frac{\left|{\mathrm{DROI}}_{\mathrm{mean}}-{\mathrm{SROI}}_{\mathrm{mean}}\right|}{\sigma }\right)$$

where DROI_mean_ is the arithmetic mean of the defective area; SROI_mean_ is the arithmetic mean of the sound area; and σ is the standard deviation of the defective area.

Figure 8 shows the CNR trend according to the modulation frequency of D_1_, A_1_, A_3_, and D_3_. CNR also showed a similar trend to phase contrast. CNR also showed a similar trend to phase contrast. The best contrast was shown at a modulation wave frequency of 0.1 Hz, and the maximum CNR value was 31.4413 dB for D_1_, 22.8425 dB for A_1_, 22.3637 dB for A_3_, and 21.9970 dB for D_3_.

Comparing the relationship between phase contrast and CNR, as shown in Table 1, it can be seen that the best CNR was obtained where the maximum phase contrast occurred (i.e., the better the phase contrast, the higher the CNR is obtained). Additionally, the closer the phase contrast is to blind frequency (0 rad), the poorer the CNR quality, and it may have a negative value. For example, the phase contrast values of the modulation frequency 0.07 Hz with negative CNR values of D_1_, A_1_, A_3_, and D_3_ were −0.0226 rad, 0.0039 rad, 0.0197 rad, and 0.0152 rad, respectively. Furthermore, at 1 Hz, they were 0.0433 rad, 0.0473 rad, −0.0089 rad, and −0.0301 rad, respectively.

### 5.3. Binary Image Evaluation

**Figure 9:**
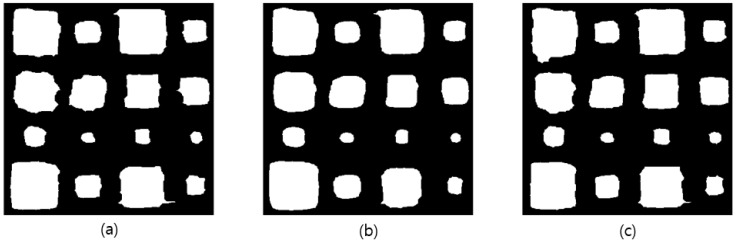
Results of binarized images through Otsu algorithm processing: (**a**) 0.05 Hz, (**b**) 0.1 Hz, (**c**) 0.2 Hz.

For water ingress detection of the phase angle images, binarization processing using the Otsu algorithm was performed on images to which median filtering was applied. Figure 9 shows the results of image binarization through the Otsu algorithm processing of Equations (7)–(12). The binarization was processed for 0.2 Hz and 0.05 Hz, which showed relatively good defect detection potential including 0.1 Hz, which showed the maximum phase contrast. The optimum threshold value for the phase angle for binarization was 4. As shown in Figure 9, all water ingress was detected at 0.05 Hz, 0.1 Hz, and 0.2 Hz. However, unlike 0.1 Hz, at 0.05 Hz and 0.2 Hz, the shape of the defect was non-uniform and very different from that of a square. It depends on the phase contrast and CNR.

The size can be estimated by the number of pixels occupied by the defect in the image. In general, to measure the size of a defect, an actual length corresponding to a unit pixel was first obtained according to the angle of view of an infrared camera using an indicator. Next, the size of the defect was calculated by multiplying the length corresponding to the unit pixel by the number of pixels occupied by the defect. The size of the defect D [mm] can be expressed as Equation (15) [39].

(15) $$D=M\times \frac{L}{P}$$

where L is the length of the indicator; P is the number of fractions corresponding to the length of the indicator; and M is the number of pixels occupied by the defect.

**Figure 10:**
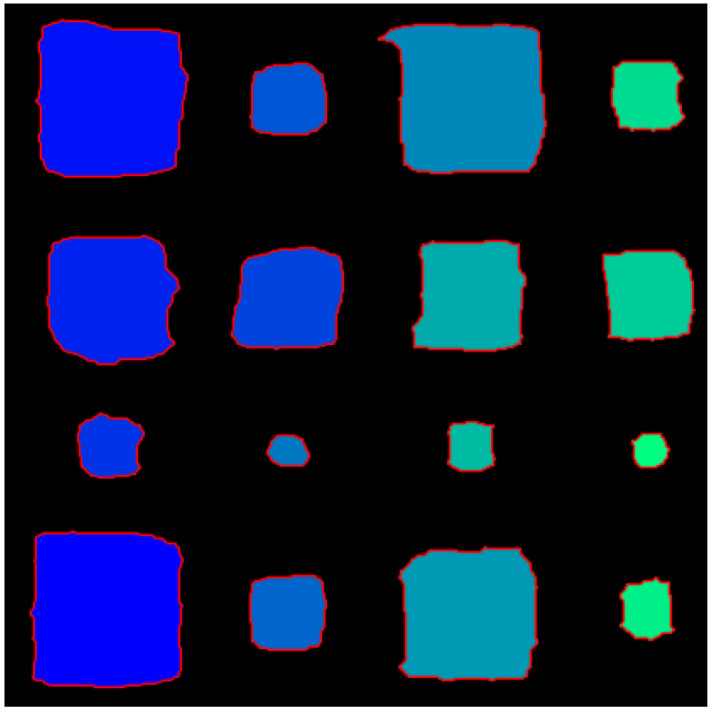
A binarized image at a modulation frequency of 0.1 Hz to calculate the number of pixels in a region.

**Figure 11:**
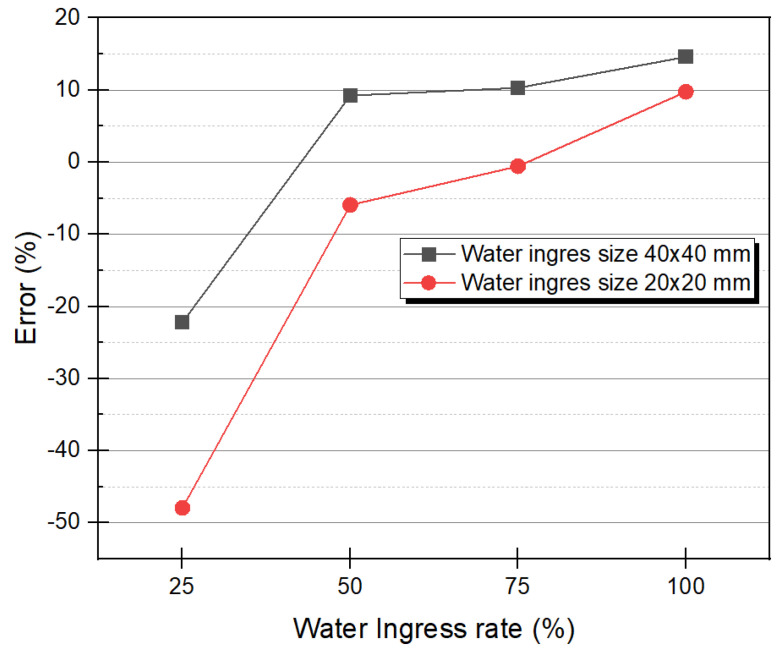
Error changes according to water ingress rate.

In order to quantitatively evaluate the binarized image, the approximate error was analyzed by comparing the number of pixels occupied by each water ingress in the image and the numerical value calculated by Equation (15). As shown in Figure 10, the number of pixels in the image was measured by counting the number of pixels representing level 1 in each area. Table 2 shows the number of pixels in the image for all water ingress and the number of pixels by theoretical calculation. For example, D1 with a water ingress rate of 100% and a size of 40 mm×40 mm was counted with 7333 pixels in the binarized image. In an actual 210 mm sample, 40 mm becomes 80 mm in a two-dimensional image of 420×420 pixels, so theoretically, it becomes 6400 pixels. Figure 11 shows the change in the measurement error of 40 mm×40 mm and 20 mm×20 mm of D_1_ and A_3_, according to the water ingress rate. In Figure 11, it can be seen that the higher the water ingress rate, the negative error changes to a positive error. This is because the higher the water ingress rate, the more active the excessive heat conduction phenomenon as external energy heating occurs on the surface and the difference in thermal conductivity between water and CFRP. Conversely, the smaller the size and the lower the water ingress rate, less heat energy is supplied and heat exchange does not occur, so the defect detection is not perfect, resulting in a negative error. For example, D_3_ with a water ingress rate of 25% had a negative error because 5078 pixels were counted in the image, 1322 pixels less than the 6400 pixels in the theoretical calculation. Moreover, in D_1_ of 100% water ingress rate, 7333 pixels were counted in the image, and there were 933 pixels more than the 6400 pixels in the theoretical calculation, so it became a positive error.

## 6. Conclusions and Future Works

This study conducted LIT experimental tests to examine the water ingress of composite honeycomb sandwich panels containing various sizes by the water ingress rate levels. Non-destructive testing by lock-in thermography reduced the effect of non-uniform heating. The main observations of this study are as follows. First, all water ingress defects under various conditions were detected by the LIT technique, and the performance of detectability was excellent. Second, the concept of LIT data processing and the Otsu algorithm for binarization were reviewed. The optimum frequency was derived by analyzing the relationship between the phase contrast and CNR according to the modulation frequency; a negative CNR value was obtained as the phase contrast was closer to 0 rad. Third, the binarization by the Otsu algorithm was evaluated for detectability by simplifying the defect shape. As a result, the binarization image by the Otsu algorithm had good detectability of water penetration, but it was greatly affected by the thermal conductivity of the material. Finally, due to the significant difference in thermal conductivity between two materials, caused by the active heat transfer on the surface, and the stronger the heat source, the greater the effect.

Through this study, it is proposed that the water ingress inside the composite material can be detected using lock-in thermography. In future studies, we will consider advanced noise removal techniques based on thermal properties. In addition, data and image processing studies will be considered for predictive simulation and experimental studies of non-linear defects such as damage and cracks. Furthermore, extension studies of LIT such as deep learning-based automated defect recognition, classification, and detection enhancement will be performed.

## Figures and Tables

**Table 1:** Quantitative data of the phase contrast and CNR according to the modulation frequency.

		Modulation Frequency (Hz)							
		1	0.5	0.2	0.1	0.07	0.05	0.03	0.01
Phase contrast (rad)	D_1_	0.0433	0.0626	0.1760	0.3502	−0.0226	0.0831	0.1368	−0.1028
	A_1_	0.0473	0.0472	0.1301	0.2247	0.0039	0.0579	0.0361	0.0800
	A_3_	−0.0089	0.0408	0.0969	0.1641	0.0197	0.0452	−0.0389	−0.0547
	D_3_	−0.0301	0.0184	0.0705	0.1288	0.0152	0.0333	−0.0433	−0.0905
CNR (dB)	D_1_	−17.1640	6.8069	21.9868	31.4413	−1.0908	22.6534	31.3556	21.3124
	A_1_	−19.8138	0.7344	15.5065	22.8425	0.8905	17.4466	20.381	22.638
	A_3_	−37.0697	1.4121	14.8205	22.3637	−6.4163	17.5077	17.4407	19.6345
	D_3_	−10.5926	−2.5889	13.6945	21.997	−20.3773	15.8543	15.8581	7.8589

**Table 2:** Estimation of the number of pixels and error for water ingress.

Water Ingress No.	Pixels		Error (%)
	Image	Theory	
A_1_	7059	6400	10.30
A_2_	1591	1600	−0.56
A_3_	6991	6400	9.23
A_4_	1505	1600	−5.93
B_1_	4400	3600	22.22
B_2_	3019	2500	20.76
B_3_	3640	3600	1.11
B_4_	2427	2500	−2.92
C_1_	1086	900	20.67
C_2_	362	400	−9.5
C_3_	753	900	−16.33
C_4_	324	400	−19.00
D_1_	7333	6400	14.58
D_2_	1756	1600	9.75
D_3_	5078	6400	−22.22
D_4_	632	1600	−47.94

## Data Availability

Due to the nature of this research, participants of this study did not agree for their data to be shared publicly and are only available upon reasonable request.
